# Research on the Preparation Parameters and Basic Properties of Premelted Calcium Aluminate Slag Prepared from Secondary Aluminum Dross

**DOI:** 10.3390/ma14195855

**Published:** 2021-10-06

**Authors:** Shaoyan Hu, Deyong Wang, Dong Hou, Wei Zhao, Xianglong Li, Tianpeng Qu, Qingde Zhu

**Affiliations:** 1 School of Iron and Steel, Soochow University, Suzhou 215137, China; syhu616@suda.edu.cn (S.H.); deyongwang1222@163.com (D.W.); xlli202005@163.com (X.L.); tianpengqu8119@163.com (T.Q.); 2 Hongxing Iron and Steel Co., Ltd., Jiuquan Iron and Steel Group, Jiayuguan 735100, China; zhuqingde@jiugang.com

**Keywords:** secondary aluminum dross, premelted calcium aluminate slag, thermodynamic calculation, lime-based calcination, leaching toxicity

## Abstract

Secondary aluminum dross is a byproduct of the electrolytic aluminum industry, whose main components are Al_2_O_3_, AlN and Na_3_AlF_6_. Secondary aluminum dross is a type of hazardous waste, with a tremendous yield every year. Realizing the harmless treatment or resource utilization of secondary aluminum dross has important economic and social benefits. In the present research, the process of preparing premelted calcium aluminate slag used for molten steel refining from secondary aluminum dross was studied in detail. Firstly, the chemical composition and phase component of secondary aluminum dross were analyzed systematically. Then, according to phase diagram analysis and melting point measurement, the appropriate mixing ratio of CaO and secondary aluminum dross and the appropriate calcination temperature were determined. On this basis, an experiment of premelted calcium aluminate slag preparation was carried out in a tubular resistance furnace. The phase component and micromorphology of the premelted slag were analyzed by XRD and SEM. The results show that the main component of the premelted calcium aluminate slag is 11CaO·7Al_2_O_3_·CaF_2_ phase with a low melting point. The original Na_3_AlF_6_ phase, which is the cause of leachable fluoride in secondary aluminum dross, disappears totally, and there is no water-soluble fluoride detected in the leaching toxicity detection. The research indicates that the process of preparing premelted calcium slag from secondary aluminum dross is feasible, which provides a helpful reference for the resource utilization of secondary aluminum dross.

## 1. Introduction

Aluminum is a nonferrous metal, with the largest production and consumption in the world [1]. It is widely used in construction, transportation, electric power, aerospace and other fields, playing an important role in the national economic construction and national defense industries as a basic raw material [2]. At present, metallic aluminum is generally produced by the process of cryolite-alumina molten salt electrolysis, which is a multiphase electrolyte system composed of cryolite-based fluoride as solvent and alumina as solute [3]. Hence, the Na_3_AlF_6_-Al_2_O_3_ binary system and Na_3_AlF_6_-AlF_3_-Al_2_O_3_ ternary system are the basis of the electrolytic aluminum industry [4,5].

Aluminum dross is slag floating on the surface of molten aluminum during the electrolytic process, which is composed of unreacted alumina, cryolite and other raw materials, as well as a small amount of other impurities generated from chemical reactions and the falling-off of anode and cathode materials [6]. It is estimated that 30–50 kg of aluminum dross is generated with the production of 1 ton of metallic aluminum. Considering the tremendous yield of metallic aluminum, millions of tons of aluminum dross are newly generated every year in the world [7]. The aluminum dross generated from the electrolytic process is generally called primary aluminum dross, which usually contains more than 50 wt.% metallic aluminum. Most of the metallic aluminum in primary aluminum dross can be easily separated and recovered by various methods, such as ash frying, ball milling, the rotary kiln process, etc. [8,9,10,11,12]. The residue after metallic aluminum extraction is called secondary aluminum dross. The main components of secondary aluminum dross are Al_2_O_3_, AlN and a small amount of metallic aluminum, in which the AlN can react with water and generate ammonia [13,14]. Moreover, secondary aluminum dross contains a small amount of fluoride and cyanide that are soluble in water [15,16]. If secondary aluminum dross is stacked or landfilled without appropriate treatment, it will cause pollution of water resources, atmosphere and soil, which cause serious harm to the environment [17]. At present, secondary aluminum dross has been included in the list of hazardous wastes [12]. Therefore, it is of great significance to achieve harmless treatment for resource utilization of secondary aluminum dross.

Due to the low content of metallic aluminum, it is not economical to extract aluminum from secondary aluminum dross. Much research on the utilization of secondary aluminum dross has been carried out, as described below. As the content of Al_2_O_3_ in secondary aluminum dross is high, mixing secondary aluminum dross with other raw materials to prepare refractories is an important utilization approach. Adeosun et al. [18] prepared refractory bricks by mixing kaolin and secondary aluminum dross and studied the specific effects of mixing ratio and sintering temperature on refractory properties. Ewais et al. [19] utilized aluminum sludge and aluminum dross to manufacture calcium aluminate cement. Aluminum sludge is a byproduct of aluminum profile processing, and it is composed of calcium aluminate hydrate, calcium carbonate and hydroxide. Mailar et al. [20] tried to replace some sintering raw materials with secondary aluminum dross to make refractory bricks. Although the performance of the final product met the relevant basic standards, its oxidation resistance property was far inferior to refractory sintered bricks without aluminum dross addition. In a subsequent study, Li et al. [21] found that reducing the content of salt impurities in secondary aluminum dross can effectively improve the oxidation resistance level of the refractories. In recent years, with the development and wide application of flocculants, using secondary aluminum dross to produce flocculants has also been studied by many researchers [22,23,24]. Shi et al. [25] prepared a polyaluminum chloride flocculant by pickling secondary aluminum dross with hydrochloric acid. The new flocculant showed good performance in removing impurities from wastewater and other waste liquid. Du et al. [26] optimized the operation method of preparing a flocculant from secondary aluminum dross, which helped to reduce the dosage of hydrochloric acid. Chao et al. [27] studied the influence of hydrochloric acid concentration, leaching temperature, leaching time and additives and obtained optimum conditions for the preparation of polyaluminum chloride by secondary aluminum dross acid leaching. Additionally, Kang et al. [28] used secondary aluminum dross as a raw material to produce low-iron aluminum sulfate by the method of co-deposition, whose product is expected to be widely used in papermaking, textile, water purification and other fields. David et al. [29] proposed a simple method with high efficiency for generating high pure hydrogen by hydrolysis in tap water of highly activated aluminum dross.

As mentioned above, many approaches to produce industrial products from secondary aluminum dross have been studied. However, few approaches have been applied to large-scale production. On the one hand, the existence of AlN and fluorine salt in secondary aluminum dross has an unstable, negative impact on the quality of industrial products. On the other hand, the consumption amount of those industrial products is too small compared with the generation amount of secondary aluminum dross, and it is difficult to solve the problem of aluminum dross accumulation.

As mentioned above, the main components of secondary aluminum dross are Al_2_O_3_ and AlN. Coincidentally, Al_2_O_3_ is also the main component of molten steel refining slag, and AlN has reducibility and deoxidation abilities, which are beneficial to molten steel desulfurization. Therefore, the application of secondary aluminum dross in molten steel refining has attracted attention and research [30]. Li et al. [31] used secondary aluminum dross to modify the composition of molten steel refining slag. Lime, fluorite and secondary aluminum dross were mixed and added into molten steel as refining slag, and as a result, fine deoxidation and desulfurization performance were achieved. Wang et al. [32] carried out an experiment to study the effect of aluminum dross on the desulphurization of pipe line steel, using secondary aluminum dross to replace aluminum oxide in the refining slag. Similarly, better desulfurization results than traditional desulfurization agents were achieved. Both the above studies verified the excellent performance of secondary aluminum dross in molten steel desulfurization. The theoretical reason for that advantage happens to be the AlN and fluoride contained in the secondary aluminum dross. AlN has reducibility and deoxidation abilities, which can reduce the oxygen content both in refining slag and molten steel, thereby improving the thermodynamic conditions of desulfurization [33]. Fluoride helps to reduce the viscosity of refining slag and improve the kinetic conditions of desulfurization [34].

In previous research on molten steel refining slag, aluminum dross was mixed with another flux and then directly added into molten steel without premelting treatment. On the one hand, it is not conducive to the rapid melting of slag. On the other hand, cryolite in aluminum dross will volatilize while adding it into molten steel and pollutes the steelmaking environment seriously, which is one of the key factors limiting its industrial application [35,36]. In addition, because secondary aluminum dross is hazardous waste, if it is not pretreated properly, its transportation and storage are subject to limitations. Therefore, the process of preparing calcium aluminate premelted slag from secondary aluminum slag is proposed in this study. Secondary aluminum dross needs to be mixed with lime firstly, then the mixture is calcinated at high temperature. During the calcination stage, Al_2_O_3_ in secondary aluminum dross reacts with CaO in lime, forming calcium aluminate with a low melting point, and those leachable, water-soluble fluorides are transformed into harmless calcium fluoride [37,38,39]. 

In order to check the feasibility of the process and find out the appropriate operating parameters, this research took industrial secondary aluminum dross generated by an aluminum electrolysis plant as raw material. Composition analysis and phase characterization of the secondary aluminum dross were carried out firstly. On this basis, thermodynamic calculation and melting point measurement were performed to obtain the appropriate material mixing ratio and calcination temperature. Based on that, lab-scale experiments preparing premelted calcium aluminate slag were carried out. Mineralogical characteristics and leaching toxicity of the premelted slag were detected.

## 2. Analysis of Secondary Aluminum Dross

As mentioned above, the secondary aluminum dross was obtained from an aluminum electrolysis plant, which was the residue left after extracting metallic aluminum from primary aluminum dross by the ash-frying method. As the key raw material of the present research, it was essential to obtain the basic properties of the secondary aluminum dross, including chemical composition and phase component. The secondary aluminum dross was ground to a particle size smaller than 0.074 mm with a ball mill pulverizing machine; then, the pulverized dross was sent for detailed inspection and analysis. Firstly, qualitative chemical composition analysis of the secondary aluminum dross was carried out by the X-ray fluorescence spectrometry method, and the results are shown in Table 1. Major elements in the secondary aluminum dross are Al and O, indicating that the main components are Al_2_O_3_ and other aluminum-containing compounds, which conform to the typical properties of secondary aluminum dross [6]. Apart from the Al and O, contents of the F, Na, Cl and Mg are relatively high, more than 1%. 

Based on the results of the qualitative analysis, a subsequent quantitative analysis was carried out to obtain an accurate chemical composition. Referring to a previous report [6] and the present qualitative analysis results, contents of the total aluminum (T.Al), metallic aluminum (M.Al), total fluorine (F), total nitrogen (N) and total sodium (Na) were detected purposefully. The results of the quantitative analysis are shown in Table 2. Comparing the detection results shown in Table 1 and Table 2, it can be seen that the deviation of the main elements is small, indicating that the detection results are accurate and acceptable. As can be seen from Table 2, the content of M.Al is only 4.58%, indicating that most of the metallic aluminum in the primary aluminum dross was extracted and recovered. 

**Figure 1:**
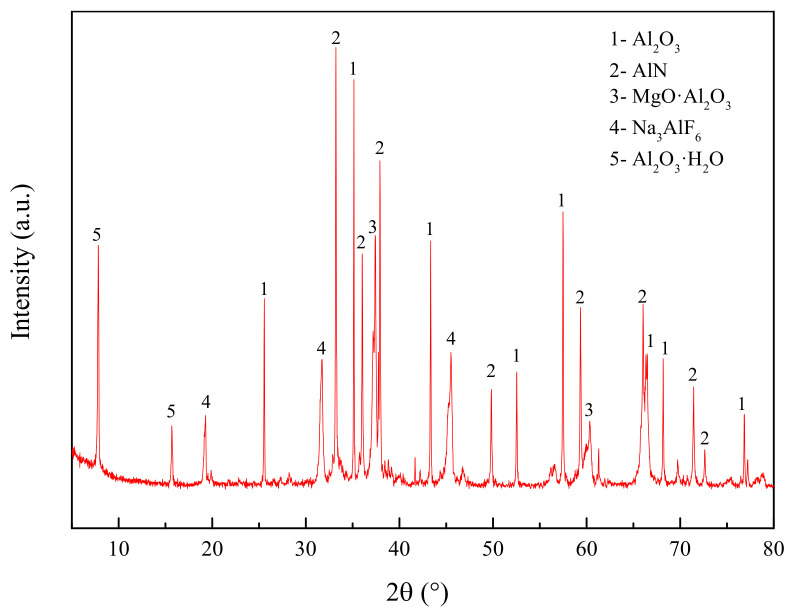
X-ray diffraction phase analysis of the secondary aluminum dross.

Both Table 1 and Table 2 show only the element contents. In order to know the specific components of the secondary aluminum dross well, it was necessary to analyze its phase component by the X-ray diffraction method (XRD). The XRD analysis result of the secondary aluminum dross is shown in Figure 1. The main phases in the secondary aluminum dross are Al_2_O_3_, AlN, MgAl_2_O_4_, Na_3_AlF_6_ and Al_2_O_3_·H_2_O. 

According to the chemical composition and phase component, it can be concluded that the element of N mainly exists in the form of AlN, the element of Mg mainly exists in the form of MgO·Al_2_O_3_ and the element of F mainly exists in the form of Na_3_AlF_6_. Based on the element contents shown in Table 2 and the phase components, the content of different phases can be estimated. It is assumed that all the N exists in the form of AlN. Since the content of N is 9.92%, the content of AlN should be 29.05%. It is assumed that all the F exists in the form of Na_3_AlF_6_. Since the content of F is 2.61%, the content of Na_3_AlF_6_ should be 4.81%. Similarly, it is assumed that all the Mg exists in the form of MgO·Al_2_O_3_. Because the content of Mg was not quantitatively detected, the content of MgO·Al_2_O_3_ was estimated based on the qualitative analysis result. Since the content of Mg is 1.34%, the content of MgO·Al_2_O_3_ should be 6.15%. Finally, it is assumed that all the rest of the aluminum elements, apart from those contained in M.Al, AlN, Na_3_AlF_6_ and MgO·Al_2_O_3_, exist in the form of Al_2_O_3_. Based on the above assumption, the content of Al_2_O_3_ could be estimated, and the calculated content of Al_2_O_3_ was found to be 54.44%.

## 3. Thermodynamic Analysis and Melting Point Measurement

The purpose of this research was to prepare premelted calcium aluminate slag from secondary aluminum dross. It was necessary to determine the appropriate proportion of lime and secondary aluminum dross, as well as the calcination temperature. At high temperature, CaO and Al_2_O_3_ can form various calcium aluminate compounds with different melting points. Finding out the appropriate proportion with a low melting point is not only beneficial to reducing the calcination temperature but also to the rapid melting of the calcium aluminate slag during the molten steel refining process. 

### 3.1. Phase Diagram Analysis

**Figure 2:**
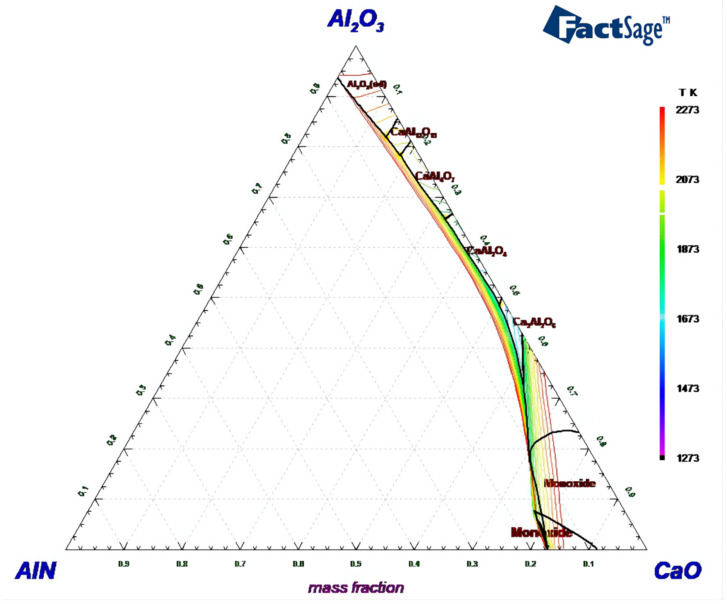
CaO-Al_2_O_3_-AlN ternary phase diagram for *p* = 1 atm, drawn with FactSage 7.2.

**Figure 3:**
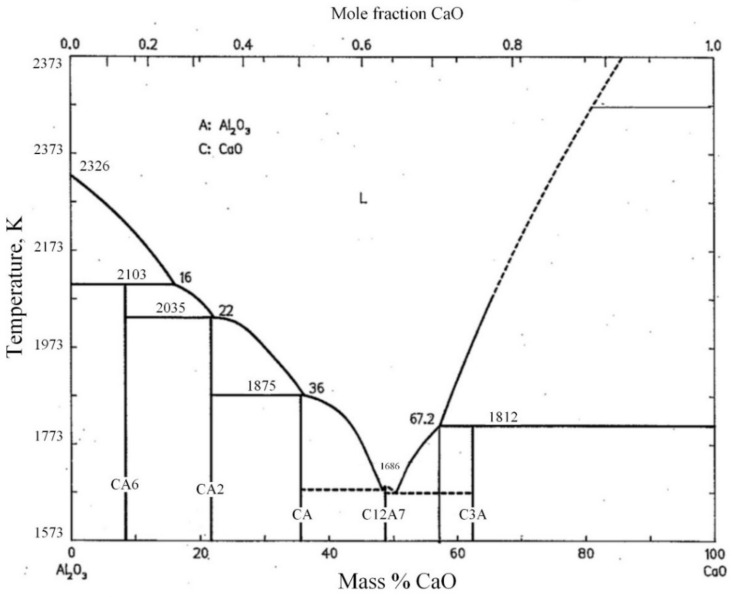
CaO-Al_2_O_3_ binary phase diagram, obtained from the slag atlas [40].

Considering that the main components of secondary aluminum dross are Al_2_O_3_ and AlN, a CaO-Al_2_O_3_-AlN ternary phase diagram was calculated and drawn and is shown in Figure 2. The ternary phase diagram was calculated and drawn by thermodynamic software FactSage 7.2, which was developed by Thermfact/CRCT (Montreal Canada) and GTT-Technology (Aachen, Germany). During the ternary phase diagram calculation, the selected database was FToxide, and the operating pressure was 101,325 Pa. Isotherm lines from 1273 K to 2273 K were plotted in the diagram with an interval of 50 K. According to the CaO-Al_2_O_3_-AlN ternary phase diagram, it is obvious that the addition of AlN will significantly increase the melting point temperature of the mixture. CaO and Al_2_O_3_ can form a low-melting point phase of calcium aluminate, while AlN can hardly form a new phase with CaO and Al_2_O_3_. The XRD analysis results shown in Figure 1 also illustrate that the AlN exists as an independent phase. Thus, in order to analyze the formation thermodynamics of calcium aluminate more clearly, a CaO-Al_2_O_3_ binary phase diagram was obtained from the slag atlas [40] and is shown in Figure 3, ignoring the influence of the AlN phase temporarily. 

As shown in Figure 3, there exist five binary phases: 3CaO·Al_2_O_3_∙(C3A), 12CaO·7Al_2_O_3_ (C12A7), CaO·Al_2_O_3_ (CA), CaO∙2Al_2_O_3_ (CA2) and CaO·6Al_2_O_3_ (CA6). The liquidus temperature drops rapidly while adding Al_2_O_3_ to CaO. The peritectic temperature between CaO and 3CaO·Al_2_O_3_ is 1812 K. The minimum melting compositions of the CaO-Al_2_O_3_ binary system are the eutectics between 12CaO∙7Al_2_O_3_ and either 3CaO∙Al_2_O_3_∙or CaO·Al_2_O_3_, which are located at 1673 K and 1668 K, respectively. Once beyond the 12CaO∙7Al_2_O_3_ eutectic with CaO∙Al_2_O_3_, the liquidus temperature begins to rise rapidly with the increase in aluminum content. The eutectic temperature between CaO·Al_2_O_3_ and CaO·2Al_2_O_3_ is 1875 K. The eutectic temperature between CaO·2Al_2_O_3_ and CaO·6Al_2_O_3_ is 2035 K. The peritectic temperature between CaO·6Al_2_O_3_ and Al_2_O_3_ is 2103 K.

**Figure 4:**
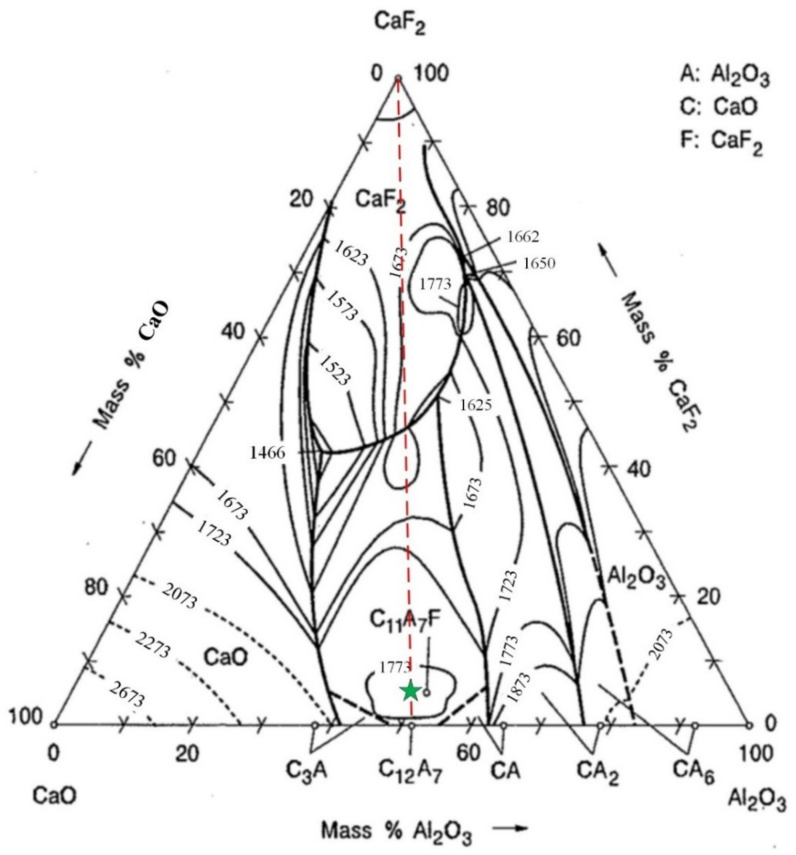
CaO-Al_2_O_3_-CaF_2_ ternary phase diagram, obtained from the slag atlas [40].

Considering the existence of Na_3_AlF_6_ in secondary aluminum dross, it is necessary to analyze the effect of Na_3_AlF_6_ on the CaO-Al_2_O_3_ system. However, there is no CaO-Al_2_O_3_-Na_3_AlF_6_ ternary phase diagram currently. As reported by Huang et al. [39], Na_3_AlF_6_ can react with CaO and Al_2_O_3_ according to the formula shown in Equation (1). Na_3_AlF_6_ can be transformed into CaF_2_ at high temperature. Due to the lack of a CaO-Al_2_O_3_-CaF_2_-Na_2_O phase diagram, the CaO-Al_2_O_3_-CaF_2_ ternary phase diagram was adopted for analyzing the influence of fluoride, as shown in Figure 4. Considering the low content of Na in secondary aluminum dross, the analysis is considered acceptable. As shown in Figure 4, adding CaF_2_ into the CaO-Al_2_O_3_ system is helpful to reduce the melting temperature. If the mixing ratio of CaO and Al_2_O_3_ is near the 12CaO·7Al_2_O_3_ phase, adding a small amount of CaF_2_ can form a new phase of 11CaO·7Al_2_O3·CaF_2_, whose melting point is around 1773 K.

17CaO + 6Al_2_O_3_ + 2Na_3_AlF_6_ = 5CaF_2_ + 3Na_2_O + 11CaO·7Al_2_O_3_·CaF_2_ (1)

As a summary, in order to obtain a low-melting point mixture of lime and secondary aluminum dross, the ratio of CaO to Al_2_O_3_ should be adjusted near to 12CaO·7Al_2_O_3_, whose mass ratio is 0.94.

### 3.2. Melting Point Measurement Experiment

#### 3.2.1. Experimental Scheme

Based on the above phase diagrams’ analysis, melting point measurement experiments were carried out to study the effect of the raw material mixing ratio. An analytical reagent of CaO was used for raw material mixing, whose CaO content was more than 98.0%. According to the analyzed composition of secondary aluminum dross, the studied mass ratio of CaO to secondary aluminum dross was changed from 0.4:1 to 1.2:1, with an interval of 0.2.

#### 3.2.2. Experimental Procedures and Devices

**Figure 5:**
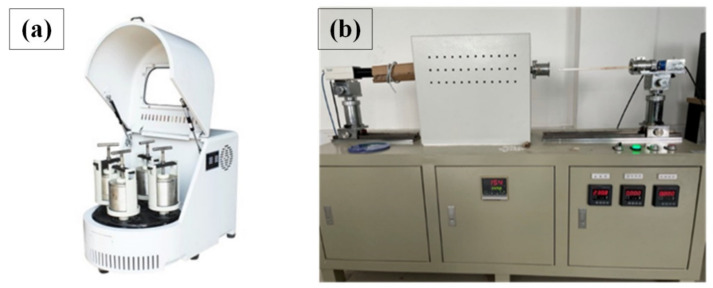
Devices used in melting point measurement experiment. (**a**) Ball milling machine; (**b**) melting point measuring instrument.

The analytical reagent of CaO and the secondary aluminum dross were mixed firstly according to the experimental scheme. Then, the mixed raw material was placed into a ball milling machine for further crushing and mixing. All of the raw materials were ground to a particle size smaller than 0.074 mm. Then, the mixed powdery raw material was pressed into a tablet and pushed into the melting point measuring instrument. The heating rate of the instrument was about 10 K/min, and the maximum temperature was about 1723 K. An image of the sample can be observed by a camera so as to judge the softening temperature, hemisphere temperature and flowing temperature of the sample. Pictures of the ball milling machine and the melting point measuring instrument are shown in Figure 5.

#### 3.2.3. Results and Discussion

**Figure 6:**
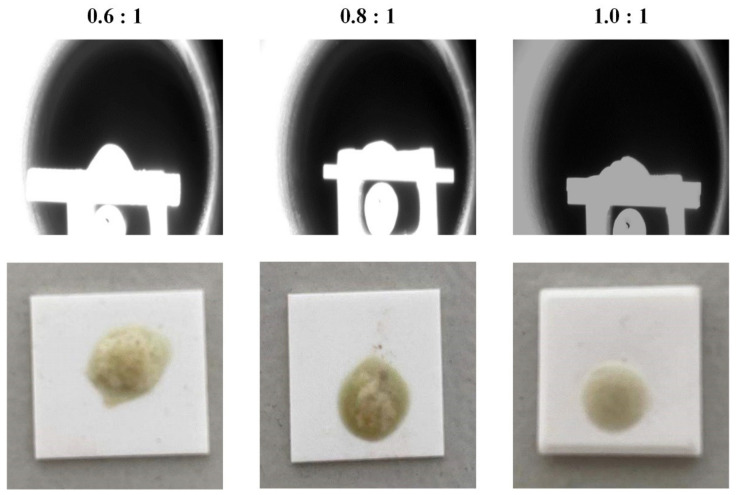
Pictures of the melted samples during melting point measurement.

Results of the melting point measurement are shown in Table 3. Within the temperature limit of the instrument, only the samples with mass ratios of 0.6:1, 0.8:1 and 1.0:1 melt. Pictures of the three samples in hemispherical shape and after being taken out from the instrument are shown in Figure 6. Other samples could not even soften. When the mass ratio of CaO to secondary aluminum dross changes from 0.6:1 to 1.0:1, the melting temperature decreases slightly. The flowing temperatures of all three samples are lower than 1723 K. The measured results indicate that as long as the mass ratio varies from 0.6:1 to 1.0:1, the flowable liquid phase can be obtained at 1723 K. Considering that the difference in melting temperature between the above three samples is small, and the mass ratio of 0.6:1 is closer to the composition of 12CaO·7Al_2_O_3_, the mass ratio of 0.6:1 and the calcination temperature of 1723 K were selected for subsequent research. 

### 3.3. Thermodynamic Calculation of the Calcination Product

In order to understand the reaction product of CaO and secondary aluminum dross at high temperature in advance, thermodynamic calculation was performed before the premelted slag preparation experiment. The reaction product was calculated by the Equilib module of FactSage software. The composition of the secondary aluminum dross was simplified as 55 wt.% Al_2_O_3_, 5 wt.% MgO·Al_2_O_3_, 30 wt.% AlN, 5 wt.% Na_3_AlF_6_, and 5 wt.% Al. As the input parameter of the Equilib module, the total weight of the secondary aluminum dross was 100 g, and the weight of CaO was 60 g. In order to compare the influence of ambient atmosphere on the reaction products, 10 g of N_2_ or 10 g of Ar was added as a reactant, respectively. Because there was fluoride in the reactants, the database of FToxid-OXFL was selected, in which CaF_2_, AlF_3_, Na_3_AlF_6_, MgF_2_ were all included. The operating pressure of the atmosphere was set as 101,325 Pa.

Results of the thermodynamic calculation are shown in Table 4. As shown by the calculation results, the reaction products contain a gas phase, liquid phase, 11CaO·7Al_2_O_3_·CaF_2_ phase, AlN phase and metallic Al. The mass proportion of 11CaO·7Al_2_O_3_·CaF_2_ in the reaction products is the largest. Phase transformations between the raw material and reaction products are discussed in detail. The Na_3_AlF_6_ phase in the original secondary aluminum dross disappears totally in the reaction products. Most of the Na_3_AlF_6_ is transformed into the 11CaO·7Al_2_O_3_·CaF_2_ phase, part of the fluoride melts into the liquid phase and little volatile fluoride is in the gas phase. The MgO·Al_2_O_3_ phase also disappears totally in the reaction products. Part of the Mg element melts into the liquid phase in the form of MgO, and the rest volatilizes into the gas phase in the form of Mg vapor. Ambient atmosphere has a big influence on the distribution of the Mg element. In N_2_ atmosphere, most of the Mg element is in the liquid phase. However, in Ar atmosphere, most of the Mg element is in the gas phase. Ambient atmosphere also has a big influence on the transformation of metallic Al. In N_2_ atmosphere, metallic Al reacts with N_2_ and forms AlN, leading to the total disappearance of metallic Al and the increase in AlN. In Ar atmosphere, metallic Al cannot be transformed into AlN, thus the weight of AlN is unchanged. However, the weight of metallic Al decreases from the initial 5 g to 3.6615 g in Ar atmosphere. This is because part of the metallic Al has a displacement reaction with MgO or CaO, which also leads to the increase in Mg and Ca vapor in the gas phase. As the main components in reactants, CaO reacts with Al_2_O_3_ to form a liquid phase and an 11CaO·7Al_2_O_3_·CaF_2_ phase, which is consistent with the analysis of the CaO-Al_2_O_3_-CaF_2_ ternary phase diagram.

## 4. Experiment of Premelted Slag Preparation

### 4.1. Experimental Scheme

**Figure 7:**
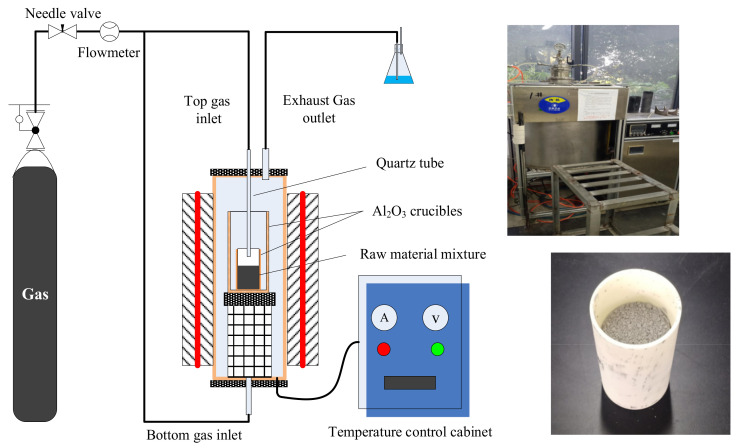
Schematic diagram and physical diagram of the experimental furnace.

Based on the selected raw material mixing ratio and calcination temperature, the experiment of premelted calcium aluminate slag preparation was carried out. The experiment was finished in a modified tubular resistance furnace, as shown in Figure 7. A gas supply device and an exhaust device were installed on the furnace. The gas supply device was used to control the ambient atmosphere in the furnace, including top blowing and bottom blowing. In the present research, N_2_ or Ar was used as a gas source to study the influence of ambient atmosphere on the calcination products, just like the condition of the thermodynamic calculation. In addition, the mass ratio of CaO to secondary aluminum dross was set to 0.6:1, the calcination temperature was set to 1723 K and the calcination time was set to 2 h. 

### 4.2. Experimental Procedures 

The experimental procedure is as follows: The analytical reagent of CaO and the secondary aluminum dross were mixed according to the mass ratio of 0.6:1. The mixed raw material was ground to a particle size smaller than 0.074 mm by a ball milling machine. Then, 200 g of the raw material mixture was placed into an alumina crucible, which was placed into the tubular resistance furnace subsequently. The furnace cover was closed, the gas supply device was turned on and N_2_ or Ar were blown according to the experimental scheme. The top blowing gas was blown through a quartz tube, the distance between whose exit and the raw material surface was set to 200 mm to avoid blowing out the powdery raw material. The flow rate of the gas was set to 10 mL/min. Then, the heating system of the furnace was turned on. The furnace started to heat up at a rate of about 10 K/min, and the temperature was maintained at 1723 K for 2 h. After that, the heating system was turned off, and the crucible cooled naturally with the furnace. Due to the thermal insulation effect of the furnace, the cooling process lasted about 12 h. After cooling to room temperature, the crucible was taken out from the furnace. The premelted slags were analyzed by XRD, SEM and leaching toxicity.

### 4.3. Results and Discussion

#### 4.3.1. XRD Analysis

**Figure 8:**
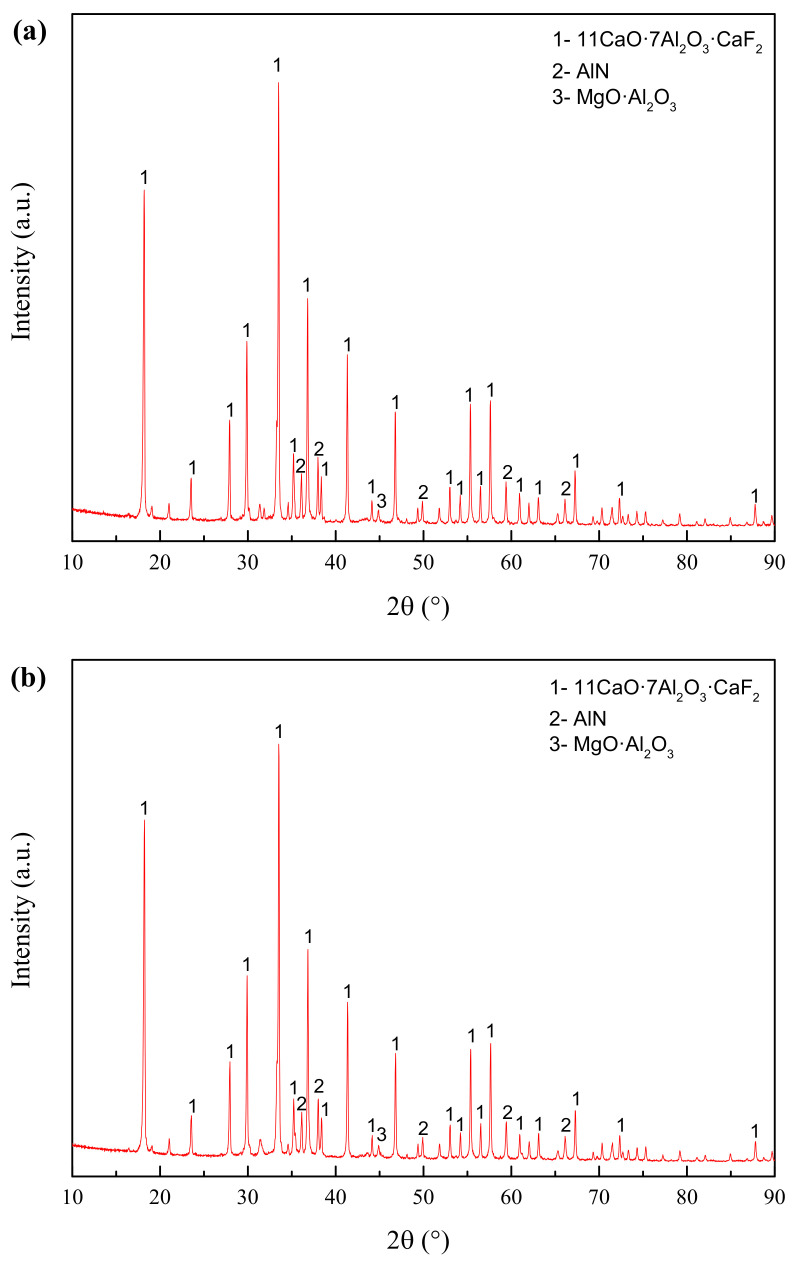
XRD patterns of the premelted calcium aluminate slags. (**a**) Calcinated in N_2_ atmosphere; (**b**) calcinated in Ar atmosphere.

Figure 8 shows the X-ray diffraction patterns of the two premelted slags. The XRD pattern of premelted slag calcinated in N_2_ atmosphere is similar with that calcinated in Ar atmosphere. The phase components in both premelted slags are 11CaO·7Al_2_O3·CaF_2_ and AlN and MgO·Al_2_O_3_, among which 11CaO·7Al_2_O_3_·CaF_2_ is the major phase. The detected phase components are consistent with the results of the thermodynamic calculation. There is a little amount of MgO·Al_2_O_3_ phase in the premelted slag, which should be formed by liquid-phase solidification during the cooling process. No metallic Al is found in the premelted slag calcinated in Ar atmosphere, which is different from the thermodynamic calculation. It is estimated that the metallic Al content may be too low to be detected, or the metallic Al may be oxidized by the residual air in the furnace. Comparing the XRD pattern of the original secondary aluminum dross shown in Figure 1 and that of premelted slags shown in Figure 8, it can be found that the Na3AlF6 phase disappears totally after calcination. All of the residual F exists in the form of 11CaO·7Al_2_O_3_·CaF_2_. 11CaO·7Al_2_O_3_·CaF_2_ has good thermal and chemical stability and is insoluble in water. The transformation of F is expected to solve the problem of leaching toxicity induced by water-soluble fluoride.

#### 4.3.2. Micromorphology Analysis

**Figure 9:**
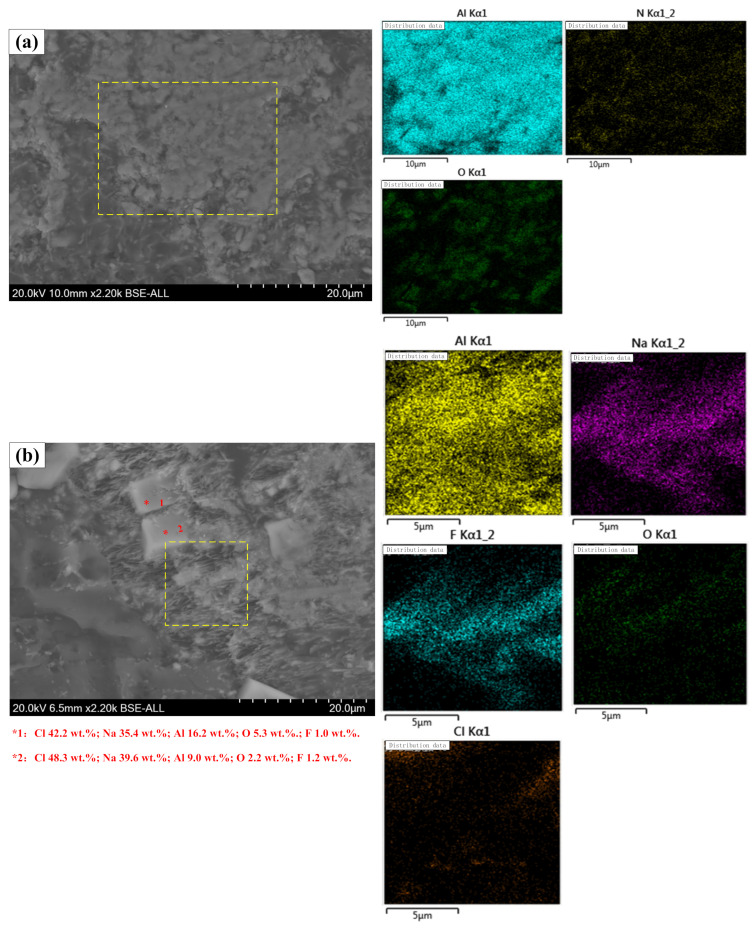
BSE micrographs of typical phases in original secondary aluminum dross, (**a**) phases of Al_2_O_3_ and AlN coexistence, (**b**) Na_3_AlF_6_ phase and NaCl phase.

Scanning electron microscope (SEM) and energy-dispersive X-ray spectrometry (EDS) were used to observe the difference of micromorphology between the original secondary aluminum dross and the premelted calcium aluminate slag. Figure 9 shows the BSE micrographs of typical phases in the original secondary aluminum dross. The morphologies of the different phases are quite different, and the distribution is uneven. As shown in Figure 9a, EDS was used to analyze the element distribution of the bright phase. According to the elemental mapping distribution, the bright phase contains Al_2_O_3_ and AlN, which coexisted and aggregated in flakes with a relatively compact structure. At the same time, a flocculent phase and bulk phase were observed in local areas, just as shown in Figure 9b. According to the elemental mapping distribution, the flocculent phase should be the Na_3_AlF_6_ phase. Two spots on the white bulk phase were selected for element analysis, the main element compositions of which are Cl and Na, indicating that the white bulk phase should be NaCl. However, there was no NaCl phase detected in the XRD pattern. It is supposed that the content of the NaCl phase is too low to be detected. From the observation of SEM, the white bulk phase is only scattered in some local areas, and the amount is very small.

**Figure 10:**
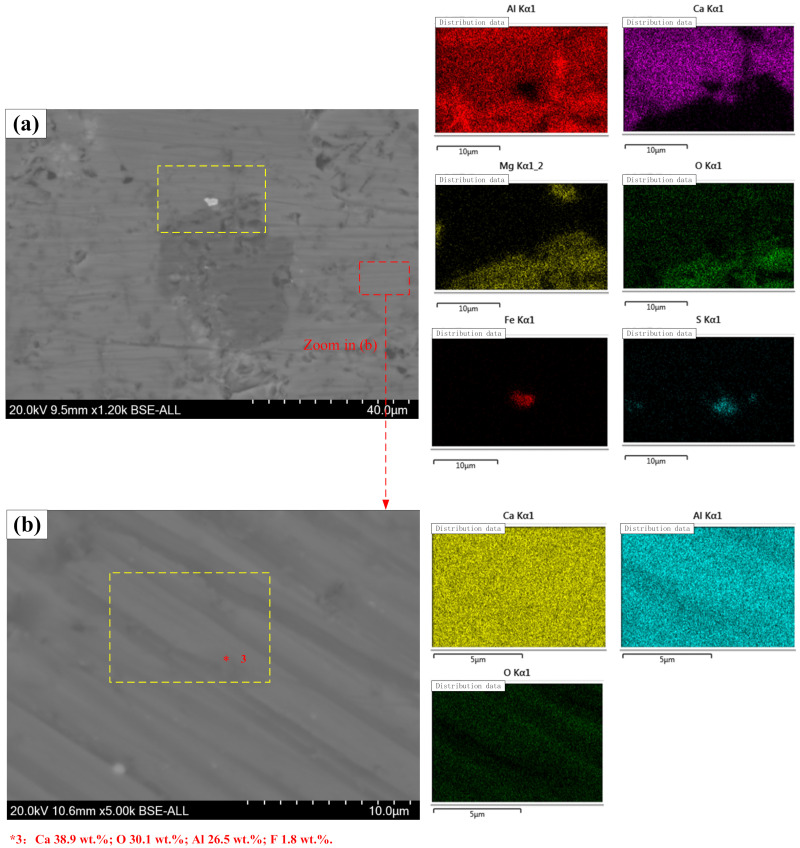
BSE micrographs of premelted calcium aluminate slag, (**a**) typical phases in slag, (**b**) enlarged view of the 11CaO·7Al_2_O_3_·CaF_2_ phase.

As a contrast, the micrographs of typical phases in premelted calcium aluminate slag are shown in Figure 10. The micromorphology of the premelted slag is more compact and uniform, mainly due to the melting process. According to the elemental mapping distribution shown in Figure 10a, the main components of the bright phase are Al-Ca-O, and the main components of the gray phase are Al-Mg-O. Thus, it is inferred that the gray phase is MgO·Al_2_O_3_. In order to reveal the accurate component of the bright phase, a magnified image of the bright phase is shown in Figure 10b. Area scan and spot scan were performed on the bright phase. The spot scan result shows that the bright phase contains 38.9 wt.% Ca, 26.5 wt.% Al and 1.8 wt.% F. According to the theoretical calculation, the 11CaO·7Al_2_O_3_·CaF_2_ phase theoretically contains 34.1 wt.% Ca, 26.8 wt.% Al and 2.7 wt.% F. Therefore, the bright phase should be 11CaO·7Al_2_O_3_·CaF_2_. Overall, the phases observed in the micrographs match well with the XRD analysis results.

#### 4.3.3. Leaching Toxicity Assessment

According to the hazardous waste identification standard in China (GB5085.3-2007), contents of the water-soluble fluoride in the original secondary aluminum dross and premelted slag were detected to evaluate their leaching toxicity. The used detection method was ion chromatography, whose detection lower limit was 0.74 mg/L. Results of the detection are shown in Table 5. “ND” in the table indicates “not detected” because the content is lower than 0.74 mg/L. It can be seen that the leachable fluoride content in original secondary aluminum dross reaches 866 mg/L, which is far higher than the national standard limit. This is the important reason why secondary aluminum dross is included in the list of hazardous wastes. Fortunately, after the calcination with CaO, the leachable fluoride content in the premelted slag is not detected, regardless of calcination in N_2_ atmosphere or in Ar atmosphere. There is almost no leachable fluoride in the premelted calcium aluminate slag prepared from secondary aluminum dross, which meets the national environmental safety standard. 

## 5. Conclusions

In the present research, the process of preparing premelted calcium aluminate slag from secondary aluminum dross was studied in detail. The appropriate mixing ratio of CaO and secondary aluminum dross and appropriate calcination temperature were determined by thermodynamic analysis and calcination experiments. The main conclusions are as follows:

1. According to quantitative chemical analysis and XRD pattern analysis, specific phase components of the used secondary aluminum dross should be: 54.44 wt.% Al_2_O_3_, 29.05 wt.% AlN, 6.15 wt.% MgO·Al_2_O_3_, 4.81 wt.% Na_3_AlF_6_, 4.58 wt.% Al.
2. Phase diagram analysis indicates that the AlN phase can hardly form a new phase with CaO and Al_2_O_3_, existing as an independent phase. The low-melting point composition of the CaO-Al_2_O_3_ binary system is near to the 12CaO·7Al_2_O_3_ phase. Adding CaF_2_ to the 12CaO·7Al_2_O_3_ phase can form a new phase of 11CaO·7Al_2_O_3_·CaF_2_.
3. When the mass ratio of CaO to secondary aluminum dross varies in the range of 0.6:1 to 1.0:1, the mixture of CaO and secondary aluminum dross can melt within 1723 K. Moreover, the melting point decreases slightly with the increase in the mass ratio in the above range.
4. Premelted calcium aluminate slag was obtained by calcinating the mixture of CaO and secondary aluminum dross with a mass ratio of 0.6:1 at 1723 K for 2 h. The premelted slag contains phases of 11CaO·7Al_2_O_3_·CaF_2_, AlN, and MgO·Al_2_O_3_, in which 11CaO·7Al_2_O_3_·CaF_2_ is the major phase. The original Na_3_AlF_6_ phase disappears completely, leading to undetectable water-soluble fluoride during the leaching toxicity detection. The experimental results agree well with the thermodynamic calculation results.

Although the composition and leaching toxicity of the premelted calcium aluminate slag meets the requirement of the molten steel refining slag, the specific metallurgical performance of the premelted slag needs further research in a follow-up study. 

## Figures and Tables

**Table 1:** Qualitative analysis of the secondary aluminum dross composition by X-ray fluorescence spectrometry.

Element	Al	O	F	Na	Mg	Cl	Si	V	Ca	S	Ti	K	Fe
Content, wt.%	50.89	23.4	3.6	2.73	1.34	1.19	0.725	0.529	0.491	0.363	0.322	0.302	0.229

**Table 2:** Quantitative analysis of the secondary aluminum dross composition.

Element	T.Al	M.Al	F	N	Na
Content, wt.%	55.49	4.58	2.61	9.92	2.25

**Table 3:** Melting point of raw material mixture measured by the experiment.

Mass Ratio of CaO to Secondary Aluminum Dross	0:1	0.4:1	0.6:1	0.8:1	1.0:1	1.2:1
Softening temperature, K	—	—	1700	1672	1653	—
Hemispherical temperature, K	—	—	1709	1686	1667	—
Flowing temperature, K	—	—	1721	1705	1699	—

**Table 4:** Thermodynamic calculation results of the CaO and secondary aluminum dross calcination product.

Phase Components	Calculation Results in N_2_ Atmosphere		Calculation Results in Ar Atmosphere	
	Weight	Specific Composition	Weight	Composition
Gas	9.3122 g	7.7302 g N_2_; 1.5469 g Na; 0.031709 g Mg; et al.	12.657 g	10 g Ar; 1.6393 g Na; 0.82349 g Mg; 0.13539 g Ca; et al.
Liq-Oxyfluoride	41.473 g	20.282 g CaO; 18.7433 g Al_2_O_3_; 1.3459 g MgO; 0.41321 g AlF_3_; 0.37792 g CaF_2_; 0.24021 g NaAlO_2_; et al.	26.643 g	13.409 g CaO; 13.039 g Al_2_O_3_; 0.071320 g AlF_3_; 0.061993 g CaF_2_; 0.050781 g MgO; et al.
11CaO·7Al_2_O_3_·CaF_2_	82.573 g	—	97.0039 g	—
AlN	36.642 g	—	30 g	—
Metallic Al	0	—	3.6615 g	—

**Table 5:** Contents of water-soluble fluoride in original secondary aluminum dross and premelted slag.

Sample	Standard Limit, mg/L	Detection Result, mg/L
Original secondary aluminum dross	100	866
Premelted slag calcinated in N_2_ atmosphere		ND
Premelted slag calcinated in Ar atmosphere		ND
